# Small Molecules and Peptides Targeting Glial Cell Line-Derived Neurotrophic Factor Receptors for the Treatment of Neurodegeneration

**DOI:** 10.3390/ijms21186575

**Published:** 2020-09-08

**Authors:** Yulia A. Sidorova, Mart Saarma

**Affiliations:** Laboratory of Molecular Neuroscience, Institute of Biotechnology, HiLIFE, University of Helsinki, Viikinkaari 5D, FI-00014 Helsinki, Finland; mart.saarma@helsinki.fi

**Keywords:** glial cell line-derived neurotrophic factor (GDNF) family ligands (GFLs), receptor tyrosine kinase Rearranged in Transfection (RET), RET agonist, GFL mimetic, small molecule, Parkinson’s disease, neuropathic pain, neurodegeneration, retinitis pigmentosa

## Abstract

Glial cell line-derived neurotrophic factor (GDNF) family ligands (GFLs) are able to promote the survival of multiple neuronal populations in the body and, therefore, hold considerable promise for disease-modifying treatments of diseases and conditions caused by neurodegeneration. Available data reveal the potential of GFLs for the therapy of Parkinson’s disease, neuropathic pain and diseases caused by retinal degeneration but, also, amyotrophic lateral sclerosis and, possibly, Alzheimer’s disease. Despite promising data collected in preclinical models, clinical translation of GFLs is yet to be conducted. The main reasons for the limited success of GFLs clinical development are the poor pharmacological characteristics of GFL proteins, such as the inability of GFLs to cross tissue barriers, poor diffusion in tissues, biphasic dose-response and activation of several receptors in the organism in different cell types, along with ethical limitations on patients’ selection in clinical trials. The development of small molecules selectively targeting particular GFL receptors with improved pharmacokinetic properties can overcome many of the difficulties and limitations associated with the clinical use of GFL proteins. The current review lists several strategies to target the GFL receptor complex with drug-like molecules, discusses their advantages, provides an overview of available chemical scaffolds and peptides able to activate GFL receptors and describes the effects of these molecules in cultured cells and animal models.

## 1. Introduction

Glial cell line-derived neurotrophic factor (GDNF) family ligands (GFLs) include three other structurally related proteins in addition to GDNF: neurturin (NRTN), artemin (ARTN, also known as enovin and neublastin) and persephin (PSPN) and, also, a distant member, growth differentiation factor-15 (GDF15) [1,2]. They all play a role in the development and maintenance of the nervous system, and GDNF is also important for kidney development and spermatogenesis [1,3,4]. GDF15 is involved in appetite control [5,6,7,8].

**Figure 1:**
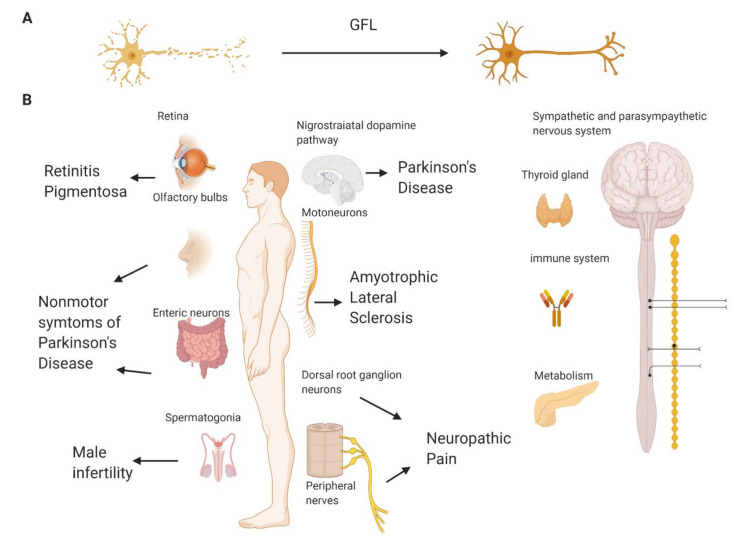
The biological effects of glial cell line-derived neurotrophic factor family ligands (GFLs) in the adult organism and potential pathological conditions that these proteins may cure. (**A**) GFLs support and restore degenerating neurons of different types. (**B**) GFLs may reduce the degeneration of retinal cells and, therefore, have potential in the treatment of retinitis pigmentosa; support the survival and guide migration of olfactory, dopamine and enteric neurons and may alleviate nonmotor and motor symptoms of Parkinson’s disease; support the survival and functioning of sensory neurons, thus reducing neuropathic pain symptoms, and are important for the survival of motoneurons, thus having the potential to alleviate amyotrophic lateral sclerosis. Outside the nervous system, glial cell line-derived neurotrophic factor (GDNF) is important for spermatogenesis and can have potential in the treatment of male infertility or the development of male contraceptives. GFLs have effects in sympathetic and parasympathetic neurons affecting multiple body functions. persephin (PSPN) and its functional receptor are expressed in the thyroid gland and has a role in thyroid cancer. Growth differentiation factor-15 (GDF15) has a role in regulating the immune system and metabolic conditions. Neurturin (NRTN) supports β cells in the pancreas, but this effect is indirect. GDNF is also important for kidney development in fetuses, but in adult organisms, its role for kidneys has not been shown. Created with BioRender.com.

The founding member of GFLs, GDNF, was discovered as a potent survival factor for dopamine neurons [9], and, therefore, it was tested in several clinical trials in patients with Parkinson’s disease (PD), as PD is characterized by the profound degeneration of dopamine neurons in the brain, resulting in motor symptoms in the disease [10,11,12,13,14,15]. The efficacy of another GFL supporting dopamine neurons, NRTN, delivered via gene therapy-based approach was also evaluated in PD patients [16,17,18]. However, the results of these clinical trials remain controversial; while, in small-scale open label ones, the positive effects of such treatments were seen, large-scale trials failed to reach their primary efficacy end point. The reasons explaining the inefficiency of GFLs in PD are reviewed in detail elsewhere [19,20] and are mainly related to the inability of these proteins to cross through the blood–brain barrier and spread into tissues. These issues make it necessary to deliver GFL proteins directly into the brain to the target region of the midbrain dopamine neurons by means of complicated and expensive stereotaxic surgery, which limits the selection of patients into clinical trials to the ones with late-stage PD. However, late-stage PD patients have very little remaining target neurons to be supported and restored by GFLs [21,22,23]. GFLs are able to support the survival of remaining neurons, regenerate their axons, restore the damaged ones (Figure 1A) and improve the functional activity of viable neurons. However, GFLs are unable to revive dead cells or produce new neurons. Therefore, the treatment with GFLs should start as soon as possible, preferably immediately upon diagnosis, which is impossible—or, at least, immensely difficult—in PD patients at the moment due to ethical restrictions on brain surgery in early stage PD patients.

The characteristic histopathological feature of PD is the presence in the brain of protein aggregates called Lewy bodies, the main component of which is alpha-synuclein [24,25,26]. Recent data indicate that the activation of GFL-dependent signaling can prevent the progression of alpha-synuclein pathology in the brain [27].

ARTN, which has a well-established function in sensory neurons, was tested in patients with neuropathic pain (NP) [28,29,30] and showed promising results in patients with painful lumbosacral radiculopathy nonresponsive to at least two standard treatments [30]. However, in this trial, the dose response to ARTN was biphasic, and the lowest dose provided the highest pain relief, while the second most efficient dose was the highest one. This kind of dose-response relationship, along with the reported side effects and potential of the ARTN to attach to extracellular matrix components, producing high point concentrations and restricting tissue spreading, complicate the clinical use of the ARTN protein.

Due to the capability to support motoneurons, GDNF has also been tested for the ability to slow down the progression of amyotrophic lateral sclerosis (ALS), an incurable disease with very poor prognosis and an average survival below five years after the diagnosis [31]. In patients suffering from ALS, motoneurons in the brain and spinal cord degenerate and die. This leads to progressive muscle atrophy and, finally, to respiratory failure, which is the most common cause of the death in ALS [32,33]. In some studies in animal models of ALS, GFLs improved the disease manifestations, although the effect could be dependent on the delivery site, concern only some symptoms and be variable in the degree of improvement [34,35,36], complicating the interpretation of the data and clinical translation. GDNF delivered to patients using stem cells was found to be safe and well-tolerated in a recent small-scale clinical trial, but the efficacy data are yet to be published [37]. Taking into account the poor diffusion of GFL proteins in the tissue [38] and widespreadness of ALS pathology, selecting the delivery paradigm for GFLs in ALS is especially difficult.

Retinitis pigmentosa is a hereditary eye disease characterized by the degeneration of retinal cells, resulting in a loss of the peripheral vision at first and blindness when the disease progresses [39]. The data on the role of GDNF in retinal cell survival are controversial, but in some studies in animal models, the positive effects of this protein were seen [40,41]. In retina, GFL receptors are expressed in Müller cells, the principal glial cells in this tissue, which have supportive and neuronal activity modulating functions [42]. GFLs delivered to an eye exert neuroprotective effects in photoreceptors indirectly via the activation of Müller cells that secrete other trophic factors necessary for the survival of retinal cells, such as fibroblast growth factor-2 [43].

GDNF also has a potential for the treatment of substance (drug) abuse and dependence via its effects in the dopamine system [44,45]. GFLs may support basal forebrain neurons [46] and promote dendrite arborization and synapse formation by hippocampal neurons [47], which degenerate in Alzheimer’s disease (AD), although proof-of-concept for this indication needs further validation. The lentiviral vector-encoded GDNF delivered into the hippocampus before the onset of symptoms preserved the memory and learning ability in a mouse model of AD [48]. However, further studies are needed to understand if the positive effects of GFLs can also be seen in mice with established AD and elucidate the mechanism of action of GFLs in this model. Human data for the conditions listed above are not yet available. GFLs exert trophic functions in the neurons belonging to autonomic nervous system (sympathetic, parasympathetic and enteric), which are important for multiple body functions, including digestion, breathing control, sweating, penile erection, etc. [4,49,50,51,52], which provides interesting opportunities for further therapeutic developments. The use of GFLs proteins in associated conditions is complicated because of their poor diffusion, but alternatives with improved properties can be considered.

Important to mention is that, in many diseases caused by neuronal degeneration or damage, neuroinflammation plays a prominent role [53,54,55]. A characteristic feature of neuroinflammation is an activation of microglial cells in the nervous system. Recent data indicate that microglial cells express GFL receptors, and GFLs can reduce microglia activation by inhibiting p38 MAPK signaling [56]. Therefore, targeting GFL receptors can also diminish the deteriorative effects of microglial cells in neurodegenerative disorders and chronic pain. Important to remember, however, is that microglial cells can play not only proinflammatory but, also, an anti-inflammatory, protective role in the nervous system, depending on their phenotype [57,58]. Further studies are needed to characterize the influence of GFLs on specific subtypes of inflammatory cells and the consequences of such effects on the tissue and organism levels.

GDF15 is aside from the other GFLs in its function. While it is important for the survival of motor, sensory and dopamine neurons [59,60,61], it plays a role in the inflammatory and cardiovascular systems, exerts nephroprotective effects and it has attracted major attention in the recent three years as a factor controlling appetite [5,6,7,8,62].

GDF15 overexpression or infusions of recombinant growth factor resulted in a decrease in food intake, improved glucose tolerance and, also, stimulated weight loss in mice fed by both standard and high-fat diets [6,63,64]. The prevalence of overweight, obesity, metabolic diseases and associated comorbidities is rising all over the world with a speed that made it be referred to as global pandemic. According to the data provided in a systematic analysis for the Global Burden of Disease Study 2013, 36.9% of adult men and 38% of women in the world have a body mass index ≥ 25 kg/m^2^ [65]. The proportion of overweight children is also rising and exceeds 10% in developed and 20% in developing countries [65]. Obesity treatments with proven efficacy include invasive gastric bypass surgery and the lipase inhibitor orlistat, which works only in a subset of patients [66]. Therefore, new noninvasive drugs to treat obesity are in high demand, and GDF15 appears as an attractive therapeutic option [67]. Interestingly, NRTN supports pancreatic β cells and, thus, may be useful in the management of diabetes [68]. This review focuses mainly on the role of GFLs in neurodegeneration; extensive data on GDF15 functions in obesity and NRTN in diabetes are provided elsewhere [5,6,7,8,62,68].

To summarize, GFLs have a clear therapeutic potential for disease modification in several diseases and conditions caused by neurodegeneration (Figure 1B). However, their clinical use is complicated because of poor pharmacokinetic characteristics, high price, variability in biological activity between batches, instability and immunogenic potential [10,13,69]. Besides, at least some neurodegenerative disorders are characterized by widespread pathologies. For example, PD patients, in addition to the degeneration of midbrain dopamine neurons, have other neuronal populations affected, e.g., enteric neurons, noradrenergic neurons, etc. In Alzheimer’s disease, neurons in several brain regions die. Sensory and motor neurons, degenerating in NP and ALS, are also located in different parts in the body. GFLs have a high binding affinity to the extracellular matrix, and therefore, their diffusion in the organism is limited [38]. Alternatives such as more diffusible neurotrophic factors, peptides and peptidomimetics, as well as small molecules targeting GFL receptors, may provide valuable tools to utilize GFLs’ potential to combat neurodegeneration and support the survival of different neuronal populations in different parts of the body. The current review will describe peptides and small molecules mimicking the biological effects of GFLs in cultured cells and animal models of relevant neurodegenerative diseases and discuss their advantages and limitations for the therapy of neurodegeneration.

## 2. Main Text

### 2.1. GFL Receptors and Signaling

**Figure 2:**
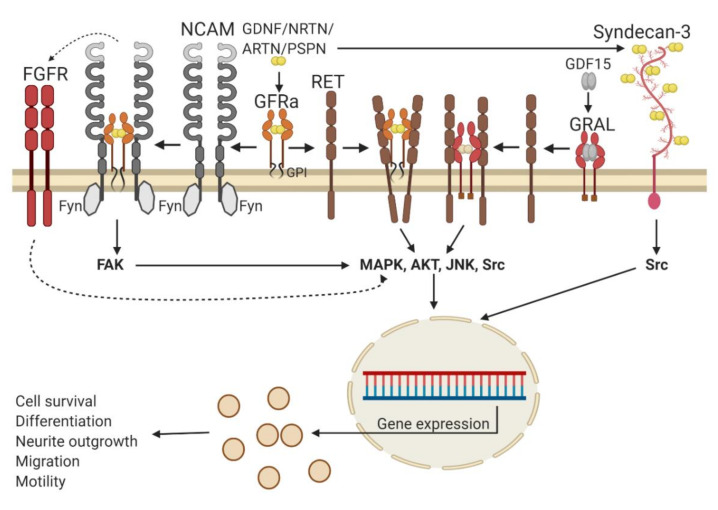
Glial cell line-derived neurotrophic factor family ligands (GFLs) signaling. The main signaling receptor for GFLs is receptor tyrosine kinase RET (REarranged in Transfection), which they activate upon binding to a glycosylphosphatidylinositol (GPI)-anchored coreceptor GDNF family receptor alpha (GFRα) or transmembrane GFRAL. RET subsequently activates classical receptor tyrosine kinase signaling pathways such as MAPK, AKT, JNK and Src, which are important for the cell survival and neurite outgrowth. In addition, GFLs in the presence of GFRα can signal via the neural adhesion molecule (NCAM) associated with the Fyn kinase, promoting cell migration and neurite outgrowth. GFLs via NCAM can also indirectly activate the fibroblast growth factor receptor (FGFR). Finally, GFLs can bind syndecan-3 and activate the Src pathway, contributing to neuronal precursor migration. Please, notice that the cognate GFRα receptor for persephin (PSPN) contains only two extracellular cysteine-rich domains. For simplification, this is not reflected in the scheme. Solid arrows indicate direct interactions and subsequent events, dotted arrows—indirect activation of certain pathways. GDNF—glial cell line-derived neurotrophic factor, NRTN—neurturin and ARTN—artemin. Created with BioRender.com.

The summary of GFL receptors and signal transduction events is presented in Figure 2. The main receptor complex transmitting GFLs signals in the organism consists of glycosylphosphatidylinositol (GPI)–anchored coreceptors GDNF family receptor alpha (GFRα1-4) or a transmembrane GDNF family receptor alpha-like protein (GRAL) and a transmembrane receptor tyrosine kinase RET (REarranged in Transfection). RET transfers signals into the cell and is shared by all five GFLs, while GFRα1-4 and GRAL function as ligand-binding subunits and are selective for individual members of GFLs. GDNF binds with the highest affinity to GFRα1, NRTN to GFRα2, ARTN to GFRα3, PSPN to GFRα4 and GDF15 to GRAL. In addition, GDNF can also interact with GFRα2 and NRTN, ARTN and PSPN with GFRα1, although with a lower affinity than with the cognate coreceptors [38]. The formation of a tripartite signaling complex that includes dimeric GFL, two molecules of GFRa or GRAL and two molecules of RET results in the phosphorylation of tyrosine residues in the RET cytoplasmic kinase domain. Phosphorylated tyrosines serve as docking sites for adapter molecules, such as Shc2 family members. Binding of adapter proteins to phosphorylated tyrosine residues in RET is followed by the activation of intracellular signaling cascades such as MAPK/ERK, PI3K/AKT, JNK and Src. These events play an important role in the survival of neurons, neurite outgrowth, neuronal migration, the differentiation of neuronal precursors and other processes important for the functioning and integrity of the nervous system [1]. In addition, an enzyme PLCγ can bind to phosphotyrosine residue 1015 in the RET kinase domain, become activated and trigger intracellular signaling events promoting the survival and neurite outgrowth, at least in certain neuronal populations [70]. The activation of RET by GDF15 via GFRAL occurs in neurons located only in two regions of the brainstem and is important for appetite control [5,6,7,8,62].

To date, it is not clear if the GFRα1/RET complex is preassembled on the cell membrane or its assembly starts from the interaction of the ligand with GFRα and the subsequent recruitment of RET [71]. Structurally, the most striking difference in the signaling complex formed by RET different ligands and coreceptors is the angle between the ligand monomers in the tripartite complex, which is the most acute in the GDF15/GRAL/RET complex and the widest in the GDNF/GFRα/RET complex [2]. The shape of the angle can influence the speed and duration of the intracellular signaling cascades’ activation [72] and, thus, fine-tunes the cellular response of individual members of the GDNF family.

In addition to RET, GFLs can also signal via the neural cell adhesion molecule (NCAM) and promote the migration of Schwann cells and neurite outgrowth from cortical and hippocampal neurons in the absence of RET, as well as influence the migration of neuronal progenitors along the rostral migratory stream [73]. NCAM is constitutively associated with the cytoplasmic Fyn kinase, which, upon stimulation, activates the focal adhesion kinase (FAK), followed by MAPK/ERK signaling cascade activation and can also activate the fibroblast growth factor receptor (FGFR) [73,74]. While GFLs can bind to NCAM itself [73,74] with a low affinity, the presence of GFRα greatly potentiates this binding and is absolutely required to trigger signal transduction in the cells [73]. Another molecule transmitting GFLs signals into cells independently of GFRα coreceptors is a heparin sulfate proteoglycan called syndecan-3. GDNF interactions with syndecan-3 activate intracellular kinase Src and stimulate neurite outgrowth from embryonic hippocampal neurons and the migration of GABAergic cortical neuronal precursors [38].

RET and GFRα coreceptors are expressed in a number of GFL responsive neurons, including dopamine, sensory, motor, sympathetic, parasympathetic and enteric neurons. GFRαs are more widely distributed in the nervous system than RET [75] and, apart from neurons, are also found in glial cells. The expression of NCAM and syndecan-3 is rather ubiquitous in the nervous system. Pleiotropic effects produced by GFLs in different cell types can result in adverse effects and complicate the analysis of clinical trial data. Therefore, the development of small molecules or peptides selectively activating certain GFL receptors or even certain signaling cascades may significantly reduce the number of side effects in patients in clinical settings.

### 2.2. Small Molecules Positively Modulating GFL Signalling

**Figure 3:**
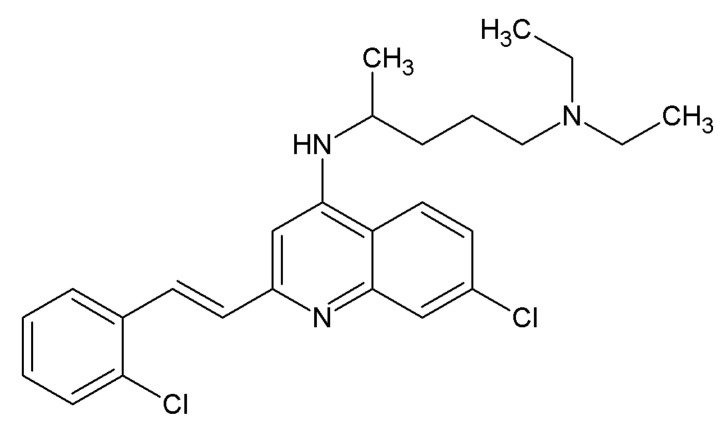
Chemical structure of the positive allosteric modulator of GFL signaling, the compound XIB4035.

The very first molecule, named XIB4035 (Figure 3), claimed to be an agonist of the GFRα1 coreceptor and was discovered by a group of scientists from Taisho Pharmaceutical Co. Ltd. and published in 2003 [76]. According to the authors, XIB4035 competed with radioactively labeled GDNF with an IC_50_ of 10.4 µM, increased RET phosphorylation at concentrations starting from 12 µM and dose-dependently promoted neurite outgrowth from Neuro-2A cells, which endogenously expressed GFRα1 coreceptor and RET [76]. The paper had a number of limitations—in particular, neither RET phosphorylation nor neurite outgrowth data were quantified and analyzed using statistical methods. Moreover, for the RET phosphorylation assay, the protein-loading control was not even presented. Cytotoxicity tests made by the authors were short-term and, therefore, not very reliable. The absence of any data on the activity of the discovered molecule in cells lacking GFRα1 and RET made it impossible to conclude on the specificity of the compound.

The follow-up paper has clearly demonstrated that XIB4035 is not actually an agonist of GFL receptors but a positive allosteric modulator of GFL signaling; this molecule increased the level of RET phosphorylation and prolonged the activation of this receptor by GDNF or ARTN, while having little, if any, biological activity towards the GFRα/RET complex in the absence of these cognate ligands. It was also demonstrated that the compound has at least some degree of selectivity towards RET, as it failed to increase the signaling induced by the nerve growth factor (NGF) or brain-derived neurotrophic factor (BDNF) via Tropomyosin receptor kinases (Trk) TrkA and TrkB, respectively. Despite the lack of truly agonistic activity towards GFRα/RET, XIB4035 efficiently prevented and reversed small-fiber neuropathy in animal models. The topical application of XIB4035 attenuated the loss of thermal nociception in a genetic model of small-fiber neuropathy caused by expression of the dominant negative mutant of the ErbB4 receptor in Schwann cells and in a streptozotocin (STZ)-induced model of diabetes mellitus (DM). Treatment with this compound also prevented the destruction of Remark bundles in sciatic nerves in ErbB4 mutant mice and reduced the loss of isolectin B4 (IB4)-binding fibers in sensory neurons in the spinal cord; however, it had no effect on the density of intraepidermal nerve fibers in diabetic animals. No significant side effects of XIB4035 in the experimental animals were reported [77].

Interestingly, some naphthoquinone derivatives, discovered recently, are also able to increase the trophic effects of GDNF in immortalized cells expressing GFRα1/RET [78]. The further optimization of such compounds could result in the development of a novel class of clinically useful positive allosteric modulators of GFL signaling.

The data presented in this section indicate that targeting RET signaling with a small molecular weight positive allosteric modulator has potential in the treatment of neuropathy [77,79]. However, the need for the presence of endogenous ligands can limit the use of such molecules to the cases with mild neuronal degeneration. XIB4035 was tested upon application to the local “affected region”, but this may be complicated in centrally manifested diseases such as PD and ALS, in which the diseased neurons may have lost connection with the regions producing GDNF. This issue can preclude the use of positive allosteric modulators for the treatment of these disorders. It was also shown that XIB4035 can increase RET signaling induced by direct RET agonists that do not require the GFRα1 coreceptor to elicit signaling [78]. This raises an interesting question of the actual target of XIB4035 or whether it can support the hypothesis of the existence of a preformed GFRα/RET complex in cells.

### 2.3. Small Molecules Targeting RET

**Figure 4:**
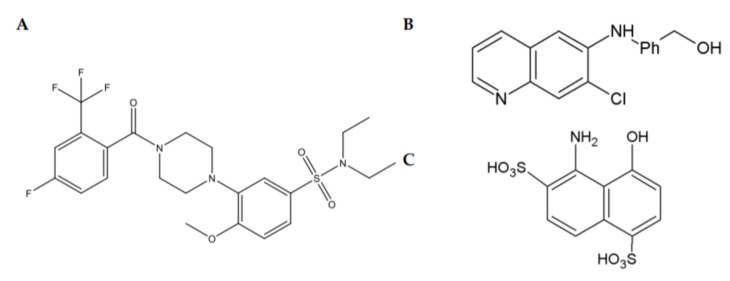
Chemical structures of RET agonists belonging to piperazine-sulfonamides (**A**); quinoline (**B**) and hydroxynaphthalene (**C**) scaffolds.

Three structurally different chemical scaffolds (piperazine-sulfonamides, quinolines and hydroxynaphthalene) targeting RET directly have been discovered in high-throughput screening campaigns (Figure 4). All of these compounds were shown to selectively increase RET phosphorylation and to activate RET-dependent intracellular signaling cascades, such as MAPK and AKT in cultured immortalized cells [78,80,81,82,83]. These compounds do not activate TrkA and TrkB or TrkA and TrkB-dependent signaling and do not support the survival of neurons lacking RET [78,80,81,82,83].

Compounds belonging to the piperazine-sulfonamide class of RET agonists, BT13 (Figure 4A) and its derivatives, BT18 and BT44, supported the survival of and/or promoted neurite outgrowth from cultured sensory neurons, alleviated hypersensitivity in animal models of neuropathic pain and protected/restored damaged sensory neurons in vivo. In healthy animals, these compounds did not influence the pain thresholds. BT13 and BT18 were tested in a spinal nerve ligation (SNL) model of neuropathic pain using a neuroprotective paradigm, e.g., a treatment with compounds was initiated immediately after the injury [83,84]. BT44 alleviated the established SNL-induced neuropathy and restored injured neurons when given to animals several days after the lesion, which more closely resembled the clinical situation than the neuroprotection paradigm. Moreover, it also reduced hypersensitivity to mechanical and cold stimuli in animals with STZ-induced DM. SNL leads to a significant reduction in the expression of sensory neuron markers, such as IB4 for nonpeptidergic neurons and the calcitonin gene-related peptide (CGRP) for peptidergic neurons located in the dorsal root ganglia. RET is expressed in the majority of IB4-positive neurons and in a portion of CGRP-positive neurons. The treatment with BT13, BT18 and BT44 protected/restored both IB4 and CGRP-positive neurons in the SNL model of neuropathic pain, similarly to ARTN [85,86] and, also, stimulated MAPK/Erk and Akt signaling in the sensory neurons [83,84]. We did not see neuronal degeneration in DM animals in our experiments, probably because, for ethical reasons, the monitoring period was rather short (max. of six weeks; afterwards, the animals had to be terminated due to poor conditions and weight loss). However, GDNF was also able to normalize the neuronal activity in sensory neurons [87], so possibly, in DM animals, BT44 worked via this mechanism [88].

BT13 and B44 also support the survival of cultured dopamine neurons RET-dependently and protect them from death induced by dopaminergic toxins, such as 1-methyl-4-phenylpyridinium and 6-hydroxydopamine [82] (Renko et al., submitted). BT13 was shown to stimulate the release of dopamine in the brain in experimental animals when given intracranially or systemically. It also activated prosurvival signaling cascades in mouse striata [82]. BT13 and BT44 alleviated motor symptoms in a rat model of PD in neuroprotective and neurorestorative paradigms, respectively. A trend to increase the density of dopamine fibers was seen in the striata of rats treated with these compounds [89]. BT compounds can cross through the blood–brain barrier with different efficacy [82,83,89] (Renko et al., submitted). BT compounds are not cytotoxic in the doses below 50–100 µM, and prohibited activity in *in vitro* CEREP screening was not manifested. These compounds were tolerated by experimental animals well. We observed no signs of acute toxicity, pain or weight loss in mice and rats treated with BT compounds systemically or intracranially. A histopathological analysis revealed that the main tissues and organs in treated animals looked normal. Using docking and molecular dynamics studies, we showed that BT compounds likely bind to the GFRα1-binding interface of RET and, thus, mimic a complex of GFRα with GFL [90].

**Figure 5:**
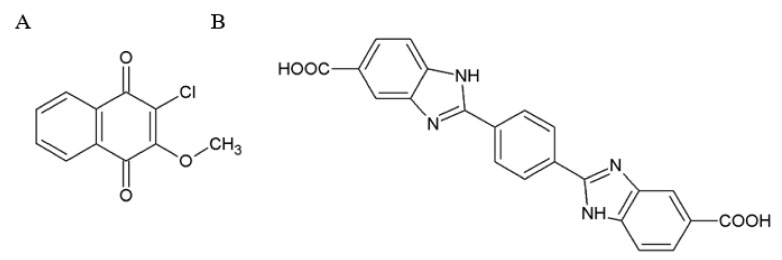
Chemical structures of GFRα/RET agonists from naphthoquinone (**A**) and benzimidazole (**B**) scaffolds.

Compounds belonging to quinoline/naphthoquinone and hydroxynaphthalene scaffolds activate intracellular signaling (both MAPK/ERK and AKT signaling pathways) in the cells expressing RET but not NGF receptor TrkA. The structure activity relationship analysis of naphthoquinone (Figure 5A) and quinoline (Figure 4B) compounds revealed the positions and chemical moieties responsible for toxicity, selectivity towards RET, potency and GFRα dependence. Hydroxynaphtalenes bind to the GFRα1/RET complex competing with GDNF (Sidorova et al., unpublished), and their biological activity can be reduced by the treatment with XIB4035. In retinal explants dissected from animals with a genetic mutation, causing retinitis pigmentosa, compounds from both of these scaffolds diminish apoptosis. When injected into the eyes of wild-type animals, these compounds activated intracellular signaling cascades in RET-expressing Müller cells [78,81]. Strikingly, in retinal explants, the efficacy of these compounds for the reduction of apoptosis exceeds that of GDNF [81], likely because tissue exposure to the GDNF protein is low because of its high affinity to the extracellular matrix [81].

### 2.4. Small Molecules Targeting GFRα Receptors

The analysis of the interacting amino acid residues in the crystal structure of the GDNF/GFRα1 complex resulted in the design of a chemical GFRα/RET agonist from the benzimidazole scaffold (Figure 5B) [91]. This compound increased the level of RET phosphorylation and activated RET signaling in the cells expressing GFRα1/RET but not RET alone. It is unknown if this compound can also target GFRα2-4/RET complexes. Although promising as GFL mimetic acting through the same receptors as GFL itself, this compound has very low biological activity and should, therefore, be substantially optimized before it or its derivatives can progress to tests in animal models of neurodegeneration [91].

Additionally, some naphthoquinones (see Section 2.3) activated RET-induced intracellular signaling GFRα1-dependently. However, these compounds also had rather high toxicity, seen already in 1–10-µM concentrations. The analysis of RET phosphorylation and intracellular signaling is conducted only with a few minutes upon compound application; subacute toxicity often does not appear in such assays. On the contrary, the analysis of neuronal survival in cultured cells and neurorestorative properties of the compounds in animal models require prolonged exposure. Therefore, naphthoquinones were not tested in primary neuron and animal models. Nevertheless, they have an interesting biological activity profile, and their further optimization directed towards reduced toxicity may result in the development of therapeutically useful molecules selective towards GFRα1.

### 2.5. Peptides Targeting NCAM and RET

**Figure 6:**
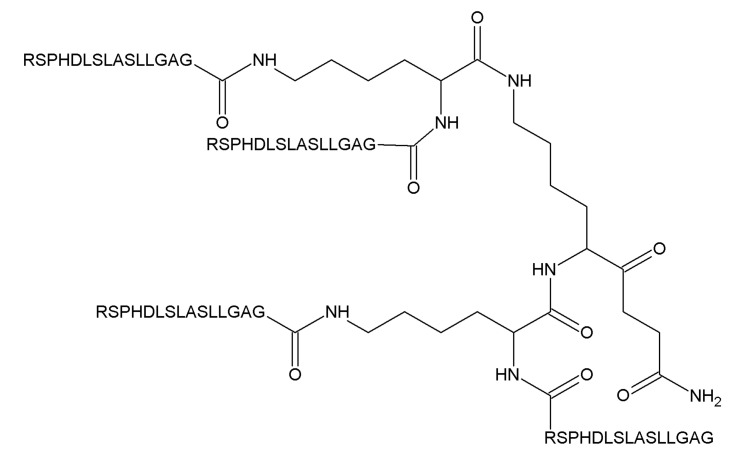
Structure of artefin.

A tetrameric peptide from the heel region of ARTN activating both NCAM and RET-dependent signaling and a peptide from the heel region of GDNF activating NCAM-dependent signaling, called artefin and gliafin, respectively, have been designed and tested in neuronal cells [74,92]. Artefin promiscuously binds to GFRα1–GFRα3 with high affinity, supports the survival of cerebellar granule neurons upon KCl challenge and stimulates neurite outgrowth from these cells [92]. Gliafin induces neurite outgrowth from hippocampal neurons that express NCAM but not RET [74]. It is not known whether it can activate RET and bind to GFRα. The data regarding the biological activity of these peptides in in vivo models are not available. It is also unknown if they can penetrate tissue barriers; they are designed as four 15-mer peptides covalently bound via a lysine backbone (Figure 6) and are, therefore, quite bulky. Artefin exhibited a survival-promoting activity in rather high concentrations within a narrow range (1.2–12.6 µM), which can also complicate the clinical translation of this peptide.

### 2.6. Peptides from the GDNF Proregion with an Unknown Mechanism of Action

A predicted endopeptidase cleavage product from the GDNF proregion called DNSP-11, a peptide that includes 11 consecutive amino acid residues from the proregion, was discovered as a molecule able to stimulate synaptic transmissions in hippocampal neurons [93]. Later, it was shown to have trophic actions in dopamine neurons in vitro and in an animal 6-hydroxydopamine (6-OHDA) model of PD [94]. An immunohistochemical analysis of rat brains revealed that this peptide, either as a free form or a part of proGDNF, can be detected in SNpc, where it colocalizes with dopamine neurons, olfactory bulbs, the cerebellum and locus coeruleus but not in the striatum. Exogenously delivered DNSP-11 is taken up by dopamine neurons in the substantia nigra pars compacta and, possibly, also by GABAergic neurons in the substantia nigra pars reticulata. DNSP-11 also supported the survival and neurite outgrowth from cultured rat embryonic dopamine neurons, reduced the number of apomorphine-induced rotations and increased the striatal dopamine content in an animal 6-OHDA model of PD. Mechanistic studies, however, revealed that DNSP-11 signals via a different receptor complex than GFRα1/RET, because it did not bind to GFRa1; its activity was not blocked by the kinase inhibitor staurosporine, and the profile of the cellular targets of DNSP-11 was different from the one seen for GDNF [94]. Importantly, DNSP-11 showed efficacy in the PD model with a severe lesion, condition in which GDNF itself often fails to produce biological effects. Therefore, this peptide can be promising for the treatment of late-stage PD patients. In this study, DNSP-11 was delivered directly into the brain, and it is unknown if it can penetrate through the blood–brain barrier and reach target neurons if injected systemically [94], but it was speculated that the systemic delivery of DNSP-11 will be unsuccessful due to a short half-life of the peptide in rat plasma (<12 min) [95]. However, it was shown that DNSP-11 retains its neuroprotective activity in the dopamine system in a 6-OHDA model of PD when delivered intranasally [95].

### 2.7. Advantages and Limitations in the Use of Each Class of GFRα/RET and NCAM Modulators for the Management of Neurodegeneration

Several classes of small molecules and peptides targeting different components of GFL-signaling complexes were described in this review. They all show promise in the treatment of neurodegeneration but all may have advantages and disadvantages for clinical use. First of all, we described here peptides and small molecules. Peptides are generally better-tolerated and are often more specific, therefore producing less adverse and off-target effects in the organism. We described here two peptides targeting GFL receptors and one produced from GDNF’s proregion [74,92,94]. Peptides targeting GFL receptors gliafin and artefin [74,92] are rather bulky, being only slightly smaller compared to GDNF itself. It is unclear if they cross the blood–brain barrier and spread in the tissues, as they have yet to be tested in vivo. At least artefin seems to target both RET and NCAM, the later molecule with widespread expression in the nervous system [92]. The side effect profile and tissue distribution of these peptides are yet to be evaluated. DNSP-11 seems to be a promising alternative for further preclinical development, as it is relatively small. The receptor and signaling mechanism for DNSP-11 is yet to be determined [94], as well as the delivery strategy, as it is unlikely to produce sufficient brain exposure upon systemic delivery due to its short half-life [95]. Possibly, it can be delivered intranasally [95].

Small molecules promoting the signaling activation or targeting components of GFL receptor complexes can spread in the tissues better than peptides and original proteins; some were shown to penetrate through the blood–brain barrier. Characterized compounds act via three different mechanisms: by promoting GFL signaling, by targeting RET directly or by targeting GFRα. Compounds promoting GFL signaling can be the most specific, as they require the presence of both endogenous ligands and full receptor complexes to elicit biological effects [76,77]. However, this feature can limit their efficacy: in many neurodegenerative disorders, neurons start to degenerate from axons, and the connection between the tissue or structure where atrophic; however viable neurons locate and target tissues or brain structures, producing the growth factor may be lost. It is also unclear if such compounds will promote only biological effects mediated by GFRα/RET or also those mediated by GFRα/NCAM.

Small molecules targeting RET are well-characterized and include structurally diverse compounds. They spread in the tissues and cross through the blood–brain barrier with different efficacy [78,81,82,83,84]. The main concern is the side effect profile, since they can activate RET in the absence of a GFRα coreceptor. RET was originally discovered as proto-oncogene, and mutated forms of RET can indeed cause cancer, mainly in the thyroid gland and adrenals [96,97]. However, it is important to remember that ligand-induced RET activation and the activation of RET when it is mutated are really different processes. Ligands activate RET transiently, which is followed by the internalization of the ligand/receptor complex and either the degradation or recycling of the receptor stripped from the ligand to limit uncontrolled signal proliferation. In addition, activation of the receptor by the ligand triggers mechanisms of signaling silencing via, e.g., the activation of phosphatases dephosphorylating the receptor [98] and other proteins in the signaling cascades. Mutated forms of RET, on the other hand, are constitutively activated, and this activation exceeds activation caused by the ligand many-fold. Furthermore, differently from wild-type RET that is activated exclusively when it is located on the cell surface plasma membrane, oncogenic RET is already permanently active in the process of protein maturation when it enters the endoplasmic reticulum, thus signaling in different cellular compartments [99]. The activation starts at the moment of embryonic development and results in cancer only after many years. Recent studies show that the overexpression of GDNF at moderate levels in mice do not stimulate tumor formation [100], supporting the idea that transient RET activation by GFL proteins or small molecular weight ligands is safe. In our own experiments with RET agonists belonging to the BT scaffold, we have not observed tumor formations (or any other serious adverse effects) in experimental animals during the monitoring period of several months [88,89] (Renko et al., submitted). Further studies are needed, however, to exclude potential off-target mutagenic and carcinogenic effects of available small molecules targeting RET.

Another potential target-related side effect of RET agonists is food intake suppression and subsequent weight loss. GDF15/GRAL/RET signaling plays an important role in appetite control [5,6,7,8], and attempts to identify druggable targets in this pathway and develop related novel treatments for obesity or cancer-induced cachexia are ongoing [62,67]. GRAL expression is restricted in the brain to the area postrema [6], where feeding centers of the brainstem are located. Interestingly, weight loss was reported as a side effect of intraventricularly delivered high doses of GDNF in a clinical trial in Parkinson’s disease patients [10]. The molecular mechanism of this GDNF action remains unresolved, as under normal conditions, GDNF does not bind to GRAL, and GRAL is required for appetite control effects mediated by GDF15/GRAL/RET [6]. The intraventricular administration of GDNF in primates and the nigral or hypothalamic overexpression of GDNF in rodents induced weight loss most probably by the activation of corticotrophin-releasing hormone neurons located specifically in the paraventricular nucleus of the hypothalamus. The activation of the hypothalamic pathway of weight control required rather high levels of GDNF overexpression [101]. When GDNF was delivered into putamen, it did not influence the weight of patients [13]; however, in this delivery paradigm, it is unlikely to reach brain regions responsible for weight gain control [38,101,102,103].

In our experiments, direct RET agonists from the piperazine-sulfonamides scaffold (BT compounds) did not influence body weights [83,88,89]. However, we either treated animals with these compounds for a relatively short time (one-to-two weeks) or delivered them intermittently. Animals were not obese and were fed by a standard non-obesogenic laboratory diet. We also noticed that the activation of GRAL/RET signaling in a reporter gene-based system [104] required rather high concentrations of recombinant GDF15 (Sidorova et al., unpublished observation). As the efficacy and potency of BT compounds are lower than that of GFLs and the compounds are rapidly metabolized, it is possible that they were insufficiently active to stimulate the hypothalamic pathway of weight gain control [102] and to promote the activation of RET in the complex with GRAL. BT compounds bind on the interface of GFRα and RET, as revealed by docking and molecular dynamics studies [90]. Therefore, it is possible that the presence of GRAL blocks their binding site in RET and, thus, precludes the activation of the weight control pathway in the brainstem.

Other scaffolds of RET agonists are yet to be tested in experiments with systemic delivery. Further studies are needed to understand the potential of RET agonists to influence food intake and weight gain in healthy and obese animals. This research can result in the development of new treatments for obesity and excessive weight. Small molecules targeting GFRα seem, at a first glance, to be more specific compared to RET. However, it is important to remember that GFRαs have a wider expression pattern in the nervous system compared to RET. In particular, a subset of sensory neurons in the dorsal root ganglia transmitting the signals from the cold stimuli express GFRα3 but not RET [105,106]. In addition, GFRα3 is also expressed in non-neuronal cells, and this may contribute to hypersensitivity to inflammatory stimuli (reviewed in [19]). In some neuronal populations, e.g., in sensory neurons, specific GFRα coreceptors are expressed only is a subset of RET-positive neurons. Therefore, compounds targeting specific coreceptors can have limited efficacy, as they will only support and rescue a small portion of lesioned neurons. Available compounds targeting GFRα either have poor biological activity or rather high cytotoxicity. They have to be thus optimized first before their efficacy in living organisms can be evaluated.

To summarize, there are several mechanisms by which GFL-related signaling can be targeted to prevent neurodegeneration. The best approach has yet to be determined. In preclinical animal models and in clinical trials, low levels of GFLs produced positive effects in the organism, eliciting neuroprotective or functional effects in certain neuronal populations in the absence of serious adverse events [14,15,100,107], while a high-level continuous overexpression or infusion produced a number of side effects [101,108]. The activation of certain neuronal populations in the body by GFLs can be beneficial for patients, as, for example, in PD, where, in addition to target nigrostriatal dopamine neurons, VTA dopamine neurons, noradrenergic neurons of the locus coeruleus and sensory, enteric and olfactory neurons (among others) are affected. This gives rise to nonmotor symptoms that are difficult to treat and impair the quality of life of the patient even more compared to motor symptoms [109]. Many neuronal populations whose dysfunction produces nonmotor symptoms express GFL receptors and can be supported by small molecules targeting them. However, GFL proteins in high doses can also produce adverse effects, e.g., the overexpression of GDNF in the hypothalamus resulted in anorexia and weight loss [101]. Therefore, it might be beneficial to limit the efficacy of developed compounds and restrict their half-life in the organism, as well as deliver them intermittently. Small molecules targeting RET are less potent and efficient than GDNF itself and do not produce such high levels of downstream signaling activation in an integral assay with luciferase readouts [82]. They are short-living compounds [82,83] that can possibly be delivered once in a few days or even weeks, as the effects of a single GDNF injection into the brain in PD models or several ARTN injections in patients made within the week can last for a long period of time (weeks or, even, months) [30]. Thus, a delivery schedule and moderate level RET activation can provide a sufficient therapeutic window to make compounds targeting RET efficient and safe for clinical use.

### 2.8. Other Possible Applications of Compounds Targeting GFL Receptors

So far, only a few compounds targeting GFL receptors have been tested in vivo in a limited number of conditions related to neurodegeneration. In view of the supportive role of GFLs in a number of other central and peripheral neurons, it is of immense interest to test developed compounds in in vivo models of different conditions and disorders, including, but not limited to, models of AD, ALS, erectile dysfunction, digestive tract motility disturbances, excessive sweating and salivation. In many of these conditions, the proof of concept with exogenously delivered GFLs is difficult to establish because of the widespread pathology and limited diffusion of GFL proteins in tissues. Small molecules or peptides, distributing widely over the body and exerting the same effects as GFLs, may represent interesting therapeutic options for the above indicated conditions.

## 3. Conclusions

Despite great promise in the treatment of neurodegenerative disorders and neuropathic pain, the clinical development of GFLs have, so far, achieved only limited success. This is due to the pharmacokinetics of these proteins, which are unable to cross tissue barriers and spread very poorly into tissues. In classic neurodegenerative disorders such as Parkinson’s disease, this means that GFL proteins have to be surgically delivered into the brain, and even in this case, they cover only a small portion of the affected tissue, as they diffuse poorly. In NP and eye diseases, these proteins can be delivered systemically or locally, respectively, but the target tissue exposure to GFL proteins remains problematic. Therapeutic efficacy issues of GFLs can be solved by the development of alternatives with better pharmacological characteristics. Several exciting molecules with improved clinical translation potentials, such as chemical compounds and peptides, targeting components of GFL receptor complexes have been described over the last two decades. At the moment, it is unknown which strategy would be the best: positive allosteric modulation of GFLs signaling or targeting RET, NCAM or GFRa coreceptors. Available molecules should be further optimized to improve their potency and other properties. Moreover, their safety has to be evaluated in extensive preclinical experiments. However, available data show that GFL receptors are druggable targets, and the modulation of their biological activity may be beneficial for the survival and well-being of retinal cells and sensory, dopamine and possibly other neuronal populations. Other major possible indications for the applications of such compounds are ALS and obesity, where in vivo proof of concept is yet to be established. Trophic effects of GFLs in the autonomic nervous system also justify testing molecules targeting GFL receptors in conditions associated with intestinal tract motility, excessive sweating and salivation, erectile dysfunction, etc. Despite promising preliminary data, many questions regarding the clinical translation of compounds targeting GFL receptors remain to be answered. In particular, the administration regimen for such compounds has to be carefully considered. Taking into account potential side effects as a result of the continuous long-term activation of RET and recent clinical trials data with intermittent delivery, we think that the best treatment paradigm will be systemic administration with regular intervals (e.g., once-monthly or weekly). However, further research is needed to best utilize the potential of GFLs and their receptors for the treatment of neurodegeneration and to establish a treatment paradigm for the developed compounds.

## 4. Patents

M.S. is an inventor in a composition of matter patent on BT compounds US 8,901,129 B2 and patent application US20200113895A1. A patent has been filed for a naphthoquinone scaffold of RET agonists on behalf of Y.A.S., M.S. and others.

## Abbreviations

AD	Alzheimer’s disease
ALS	Amyotrophic lateral sclerosis
ARTN	Artemin
BDNF	Brain-derived neurotrophic factor
CEREP	A panel of assays to evaluate potential toxicity of compounds to major systems and organs
CGRP	Calcitonin gene-related peptide
DM	Diabetes mellitus
FAK	Focal adhesion kinase
FGFR	Fibroblast growth factor receptor
GDNF	Glial cell line-derived neurotrophic factor
GFLs	Glial cell line-derived neurotrophic factor family ligands
GFRa	Glial cell line-derived neurotrophic factor family receptor alpha
GRAL	GDNF family receptor alpha-like protein
GDF15	Growth differentiation factor-15
IB4	Isolectin B4
NCAM	Neural cell adhesion molecule
NGF	Nerve growth factor
NP	Neuropathic pain
NRTN	Neurturin
PD	Parkinson’s disease
PSPN	Persephin
RET	REarranged in Transfection
SNL	Spinal nerve ligation
Trk	Tropomyosin receptor kinases
